# Topography and Corrosion Resistance Characteristics of Fe40Al5Cr0.2ZrB Alloy and X18CrN28 Steel

**DOI:** 10.3390/ma18235465

**Published:** 2025-12-04

**Authors:** Janusz Cebulski, Dorota Pasek, Stanisław Roskosz, Magdalena Popczyk, Jadwiga Gabor, Sebastian Stach, Roman Wrzalik, Marcin Wojtyniak, Michał Simlot, Andrzej S. Swinarew

**Affiliations:** 1 Department of Materials Technology, Faculty of Materials Engineering and Industrial Digitalization, Silesian University of Technology, Krasińskiego 8, 40-019 Katowice, Poland; 2 Promobil S.C., Kopernika 12, 40-064 Katowice, Poland; dorota.pasek@promobil.pl; 3 Faculty of Science and Technology, University of Silesia in Katowice, 75 Pułku Piechoty 1A, 41-500 Chorzów, Polandroman.wrzalik@us.edu.pl (R.W.);; 4 Faculty of Science and Technology, University of Silesia in Katowice, Będzińska 39, 41-200 Sosnowiec, Poland; sebastian.stach@us.edu.pl; 5 Institute of Physics, Silesian University of Technology, Konarskiego 22B, 44-100 Gliwice, Poland; 6 Department of Laryngology, Faculty of Medical Sciences in Katowice, SPSK im. A. Mielęckiego SUM, Medical University of Silesia, Francuska 20/24, 40-027 Katowice, Poland; michalsimlot@gmail.com; 7 Department of Training and Nutrition in Sports, Faculty of Physical Education, The Jerzy Kukuczka Academy of Physical Education, Mikołowska 72A, 40-065 Katowice, Poland

**Keywords:** corrosion resistance, intermetallic phases, Fe40Al5Cr0.2ZrB alloy, X18CrN28 ferritic stainless steel, electrochemical impedance spectroscopy, chloride-induced attack

## Abstract

This paper presents the results of corrosion resistance tests of materials (Fe40Al5Cr0.2ZrB alloy and X18CrN28 steel) in a 5% NaCl solution at room temperature using electrochemical impedance spectroscopy (EIS) and potentiodynamic polarization, complemented by confocal/AFM topography and SEM/EDS analysis. Confocal/AFM mapping showed pronounced roughening and localized features on Fe40Al5Cr0.2ZrB alloy (e.g., S_a_ rising locally to ~1.63 μm), consistent with heterogeneous chloride-induced attack, whereas X18CrN28 steel exhibited only minor roughness changes (S_a_ ~ 13–19 nm). SEM/EDS of Fe40Al5Cr0.2ZrB alloy revealed mixed oxides with detectable chlorine at corroded sites, while the steel retained a thin, Cr-rich passive layer with negligible Cl signal. Overall, X18CrN28 steel demonstrates significantly higher resistance to localized corrosion in neutral chloride media than Fe40Al5Cr0.2ZrB alloy, aligning electrochemical metrics with surface and chemical analyses.

## 1. Introduction

The development of heat-resistant materials engineering is closely linked to many fields of technology, primarily thermal and nuclear power generation, as well as aviation and the construction of technological equipment. However, the primary consumer of heat-resistant steels and alloys is the thermal power industry, and therefore, its development is largely dependent on commercially available materials and technologies that meet increasing quality requirements. The requirements for steels operating at elevated temperatures significantly exceed the standard characteristics used in structural calculations [1].

For many years, the basic heat-resistant materials have been metal alloys, primarily steels for use at elevated temperatures and nickel–cobalt alloys for use at high temperatures. Among all construction materials, heat-resistant steels designed for use at high temperatures and in corrosive environments have played an important role, primarily due to the development of the energy and chemical industries [2]. In addition to the required heat-resistant properties, it is important that these materials are also resistant to electrochemical corrosion. This is particularly important when the structural components of an industrial installation operate in a cyclic manner. Lowering the operating temperature of a system operating in an aggressive environment can cause the release of acid and salt compounds, which can negatively affect the surface of the materials from which they are made. It is essential to assess the corrosion resistance of materials under cyclic operation, such as during the temporary shutdown of specific installation components. Furthermore, it is worth noting that conventional coolers and heat exchangers are typically cooled with brine, such as sodium chloride (NaCl). In the case of geothermal power plants and salt installations, the pipelines are immersed in an environment with a concentration of 5–25% NaCl.

Manufacturers of stainless, acid-resistant, and heat-resistant steel products rely on several basic grades of steel, which share a common characteristic: a high content of alloying elements, including nickel, chromium, and molybdenum, as well as tungsten, vanadium, and titanium. The most used steels include ferritic, martensitic, duplex, and austenitic steels with a reduced nickel content. Selection of a specific grade must be based on an analysis of the actual operating conditions of a given product and the operational requirements that must be met. Manufacturers are increasingly seeking alternative grades to conventional steels, which will reduce costs while maintaining the same material properties.

Based on the results of research conducted on material selection and appropriate technological processes, it has been demonstrated that a matrix of iron, nickel, and titanium compounds with aluminum can create a different group of structural alloys with specific mechanical properties and a stable structure at high temperatures. Materials tested in the present comparative study were Fe40Al5Cr0.2ZrB (intermetallic, homogenized at 1050 °C for 72 h) and X18CrN28 ferritic stainless steel. Electrochemical testing in 5% NaCl, confocal and AFM surface roughness mapping, and SEM/EDS investigations were conducted to provide information on passive-film quality, localized attack, and corrosion kinetics.

Intermetallic iron–aluminum (Fe–Al) alloys based on the FeAl (B2) phase are attractive for high-temperature structural applications as they combine low density, good oxidation resistance, and favorable high-temperature strength in oxidizing atmospheres [3,4,5], with their dominant corrosion protection mechanism being the selective formation of a continuous α-Al_2_O_3_ (alumina) scale that limits further inward oxygen diffusion and outward metal transport [3,6,7]. The scale adherence, growth kinetics, and in-service mechanical behavior are strongly influenced by alloying additions such as Cr, Zr, Ti, and B, as well as microstructural control through homogenization, grain size, and dispersoids [5,8,9]. In contrast, the ferritic stainless steel X18CrN28 (EN 1.4749), which is comparable to the AISI 446 family with a high Cr content of approximately 23–28 wt.%, is designed as a heat-resisting alloy where corrosion resistance arises primarily from chromium-rich passive films (Cr_2_O_3_ and mixed Fe–Cr oxides) combined with a thermally stable ferritic matrix [10,11,12], and where nitrogen additions may further increase strength and influence passivity by modifying the matrix chemistry [10].

At elevated temperatures in oxidizing atmospheres, Fe–Al intermetallics form alumina scales via the selective outward diffusion of Al and inward diffusion of oxygen, a process that yields a slow, parabolic growth rate of dense α-Al_2_O_3_ when aluminum activity and scale continuity are sufficient [3,6,7]. This alumina scale is thermodynamically very stable and exhibits low ionic conductivity, which accounts for the excellent oxidation resistance of properly formulated Fe–Al alloys in dry, oxygen-rich environments [3,7,13]. Chromium-bearing ferritic steels, however, form chromium oxide (Cr_2_O_3_) and mixed spinel (FeCr_2_O_4_) scale layers when exposed to similar conditions; these scales are generally thinner than alumina but provide effective passivation at typical service temperatures for heat-resisting steels, with their protective character depending on chromium availability at the metal/oxide interface, scale continuity, and the absence of deleterious contaminants like sulfur or alkali chlorides that promote breakdown [10,11,12].

Although α-Al_2_O_3_ is highly protective in dry oxygen, chloride species are particularly aggressive to alumina-forming systems, where under chlorine-containing atmospheres or in aqueous chloride solutions, the alumina scales can be undermined by chloride adsorption, penetration at defects, or the formation of volatile aluminum oxy-chlorides, leading to scale thinning, local aluminum depletion, and the initiation of localized attack such as pitting [14,15,16,17]. Atomistic and experimental studies confirm that chloride ions interact with hydroxylated alumina surfaces and concentrate at defects and grain boundaries, thereby lowering the barrier to localized breakdown [15,16]. Consequently, while Fe–Al intermetallics show excellent performance in high-temperature oxidative environments, they are comparatively susceptible to localized degradation in the presence of chlorides, especially when the oxide scale is non-uniform, defective, or subjected to mechanical stresses from thermal cycling that promote spallation [5,14,17], with several studies on Fe40Al-based alloys reporting mixed behavior—protective alumina formation under benign conditions but localized or accelerated attack when halides are present or the scale is damaged [5,8,18].

Ferritic high-Cr steels, such as X18CrN28, on the other hand, develop a chromium-enriched passive film in aqueous chloride solutions that is typically more resistant to chloride penetration than the defective alumina films on some Fe-Al alloys. The favorable thermodynamics and kinetics of Cr_2_O_3_ formation, coupled with the rapid repassivation ability of chromium-containing steels, generally provide superior resistance to localized electrochemical attack in neutral chloride solutions such as NaCl when compared to many alumina-forming intermetallics under identical conditions [11,19,20], a conclusion supported by recent comparative studies indicating that grades like AISI 446 exhibit relatively noble corrosion potentials, low passive current densities, and good stability in chloride environments [20].

Electrochemical impedance spectroscopy and potentiodynamic polarization measurements in 5% NaCl typically reflect these differences: the Fe40Al5Cr0.2ZrB intermetallic exhibits a more negative corrosion potential and higher corrosion current density, indicating less robust passivation in the presence of Cl^−^, along with lower polarization resistance when localized film defects allow chloride ingress, and surface microscopy often reveals mixed oxide/hydroxide products with localized pits and oxide accumulation in corroded regions [5,16,18]. Conversely, the X18CrN28 ferritic steel demonstrates a more noble corrosion potential, substantially lower corrosion current density, and larger polarization resistance, consistent with a continuous chrome-rich passive layer, while SEM/EDS analysis typically shows thin, continuous Cr_2_O_3_/Fe–Cr oxide layers with little evidence of chloride accumulation at the film/metal interface [19,20], trends that are consistent with prior literature comparing these material classes in chloride-rich environments [5,14,20].

The fundamental mechanisms of protection and failure further elucidate this behavior. The dense, slow-growing alumina (α-Al_2_O_3_) on Fe–Al alloys under dry oxidizing conditions fails in chloride environments through mechanisms such as chloride adsorption and penetration at hydroxylated or defective surfaces, the formation of localized oxy-chloride species, mechanical spallation during thermal cycling, and local aluminum depletion that prevents the re-formation of a protective scale [14,15,16,17]. In contrast, chromia (Cr_2_O_3_) on X18CrN28 forms an inner, chromium-enriched oxide that passivates by limiting iron oxidation and promoting self-healing after local damage. This oxide exhibits good stability against chloride attack in many aqueous environments because chromium tends to re-passivate, and the thermochemistry of volatile Cr-chlorides is less favorable under typical service conditions. However, it can still be undermined in strongly acidic or highly oxidizing chloride media or by molten chlorides at high temperatures [2].

From a practical standpoint, for components exposed to dry, high-temperature oxidizing atmospheres without significant chloride contamination, Fe–Al intermetallics are attractive due to the stable α-Al_2_O_3_ scale they form. However, for aqueous chloride environments or service involving cyclic exposure to chloride-bearing media, such as cooling waters or saline spray, high-Cr ferritic steels, like X18CrN28, generally offer more reliable passive protection and a lower propensity for localized chloride-induced breakdown. In hybrid or uncertain environments, potential solutions include using protective coatings, employing local alloying strategies like increasing the Cr content in Fe–Al alloys, or applying various barrier treatments to prevent chloride access.

## 2. Materials and Methods

The tests were performed on samples of the Fe40Al5Cr0.2ZrB intermetallic alloy after casting and steel X18CrN28. The materials of the Fe40Al5Cr0.2ZrB intermetallic alloy were subjected to homogenizing annealing at 1050 °C for 72 h, homogenizing the chemical composition of the alloy. The chemical composition was presented in Table 1 and Table 2.

The primary objective of the study was to assess the corrosion resistance of the Fe40Al5Cr0.2ZrB alloy (sample 1) and X18CrN28 steel (sample 2) in a 5% NaCl solution.

The research program comprised the following steps:

1. Corrosion resistance of the Fe40Al5Cr0.2ZrB alloys and X18CrN28 steel was investigated in 5% NaCl solution using AUTOLAB^®^ system (PGSTAT30, Metrohm Autolab B.V., Utrecht, The Netherlands). A platinum mesh served as the counter electrode, and a saturated calomel electrode (SCE) was the reference electrode. These tests included the open-circuit potential measurement within 20 h, electrochemical impedance spectroscopy, and potentiodynamic polarization. EIS studies were performed at the open-circuit potential (frequency range from 50 kHz to 0.01 Hz (10 points per decade), amplitude of the ac signal 10 mV, room temperature). Potentiodynamic curves were registered within a potential range of ±250 mV relative to the open-circuit potential at a rate of 1 mV·s^−1^.
2. 3D confocal surface topography maps were obtained by an Olympus LEXT OLS-4000 confocal scanning microscope (Olympus Corp., Tokyo, Japan), and MountainsMap^®^ Premium 6.2 software.
3. AFM—Measurements were performed using the intermittent contact method. Sample topography was recorded on 10 × 10 µm surfaces. Images were processed using standard Gwyd-dion 2.63 software (align rows, mean plane subtraction, shift minimum data to zero). Roughness parameters Sq (Root Mean Square Roughness) and Sa (Arithmetic Mean Height) were determined.
4. The surface appearance of the samples following corrosion tests was examined using scanning electron microscopy (SEM) with a Hitachi S-3400 (Tokyo, Japan). The chemical composition of oxidation products on the solid samples was analyzed using an energy dispersive X-ray spectrometer (EDS) by ThermoNoran (System Seven) (Waltham, MA, USA), operated at an electron beam accelerating voltage of 15 keV. The spectrometer connects to the microscope.

**Figure 1:**
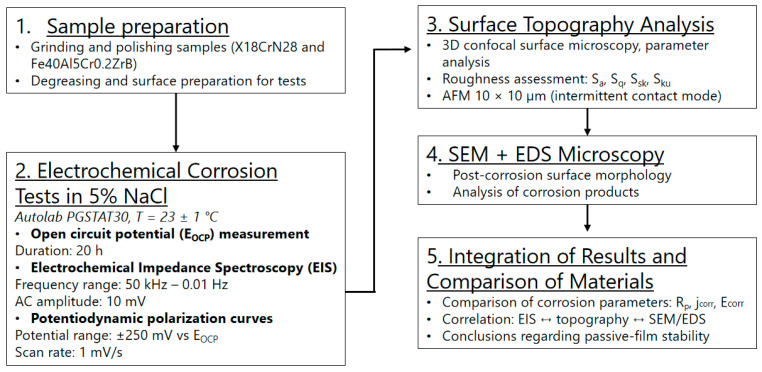
Schematic diagram of the experimental procedure.

The experimental procedure is schematically illustrated in the diagram shown in Figure 1.

## 3. Results

### 3.1. Corrosion Resistance Research

**Figure 2:**
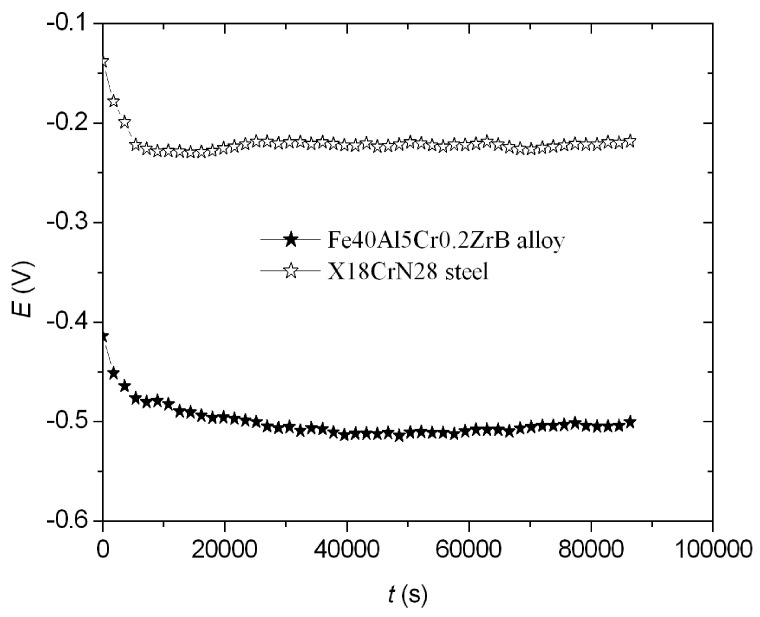
Dependences of *E* = f (*t*) for the Fe40Al5Cr0.2ZrB alloy and X18CrN28 steel.

The open-circuit potential (*E*_OCP_) of the Fe40Al5Cr0.2ZrB alloy and X18CrN28 steel after 20 h was determined (Figure 2). This potential (*E*_OCP_) was −0.500 V for the Fe40Al5Cr0.2ZrB alloy and −0.218 V for the X18CrN28 steel. In the case of the tested materials, this potential remained practically unchanged after 20 h, indicating the high stability of these materials (Figure 2).

**Figure 3:**
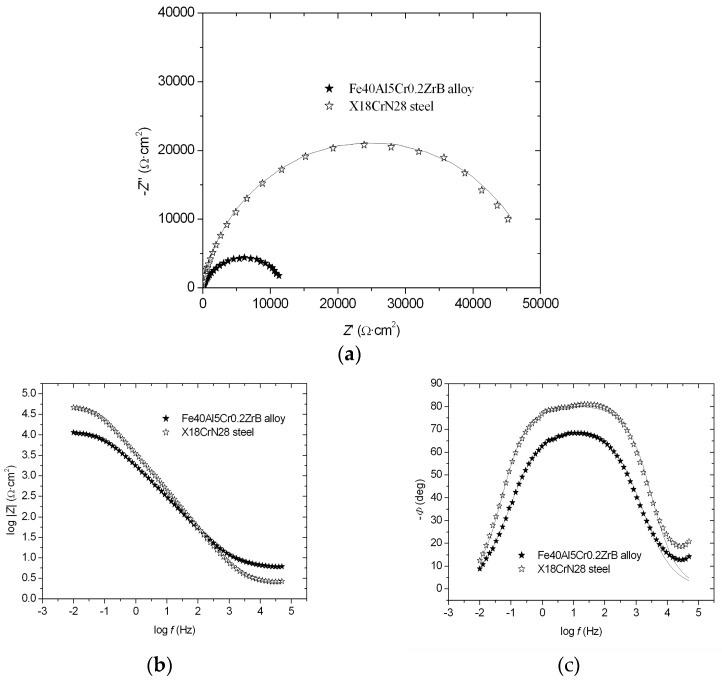
Nyquist and Bode diagrams: −Z″ = f (Z′) (**a**), log |*Z*| = f (log *f*) (**b**), and −*Φ* = f (log *f*) (**c**), registered in 5% NaCl solution for the Fe40Al5Cr0.2ZrB alloy and X18CrN28 steel (symbols—experimental points, solid lines—approximations using the one-CPE model).

The EIS results are presented in the form of Nyquist and Bode diagrams (−Z″ = f (Z′) (a), log |Z| = f (log *f*) (b), and −*Φ* = f (log *f*) (c)) (Figure 3a–c). For tested materials, one semicircle is visible over the entire frequency range (Figure 3a). It was ascertained that this behavior of the tested materials could be described by a one-CPE electrode model (as previously discussed in detail [21,22,23]). The Nyquist diagrams show a good agreement between the experimental points and approximations. Approximations of the experimental impedances permitted determinations of the following parameters: *R*_p_, *R*_s_, *T*, *ϕ*, where *R*_p_ is polarization resistance, *R*_s_ is solution resistance, *T* is the capacity parameter, and *ϕ* is the CPE angle. Based on these results, it was found that the X18CrN28 steel exhibited greater resistance to corrosion, as evidenced by a higher polarization resistance value and a lower capacitance parameter value (Table 3).

The low-frequency impedance modulus is often used as a simple index for comparing materials [18]. For example, the log|Z|_0.1_ parameter (at a frequency of 0.1 Hz) for X18CrN28 steel showed a higher value (4.32 Ω·cm^2^) compared to the Fe40Al5Cr0.2ZrB alloy (3.84 Ω·cm^2^) (Figure 3b), indicating a more effective corrosion resistance of X18CrN28 steel. Furthermore, the dependence of −*Φ* = f (log *f*) for X18CrN28 steel showed a wider range of phase angle independence from the logarithm of frequency compared to the Fe40Al5Cr0.2ZrB alloy (Figure 3c). This wider range indicates a greater corrosion resistance of X18CrN28 steel.

**Figure 4:**
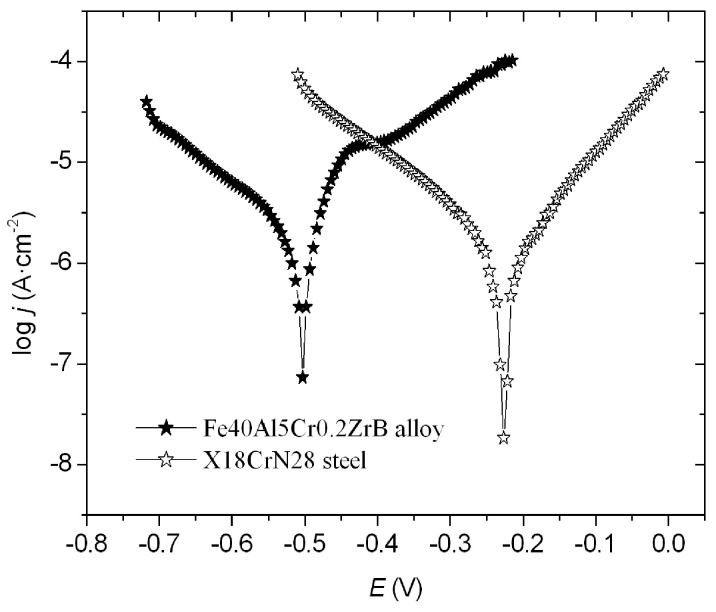
Potentiodynamic curves registered for the Fe40Al5Cr0.2ZrB alloy and X18CrN28 steel.

The corrosion resistance parameters *E*_corr_, *j*_corr,_ and *R*_p_ (Table 4) were determined based on the obtained relationship log *j* = f (*E*) (Figure 4). The obtained results show that X18CrN28 steel exhibits higher corrosion resistance compared to the Fe40Al5Cr0.2ZrB alloy. Proof of this is higher polarization resistance *R*_p_, lower current density, and a shift in corrosion potential toward positive values (Figure 4). It is worth noting that the corrosion potential does not differ significantly from the previously determined open-circuit potential (*E*_OCP_). The parameters determined by the potentiodynamic measurements allow for the estimation of the corrosion resistance of coatings and confirm the results obtained from the EIS method.

### 3.2. Research Surface Topography

**Figure 5:**
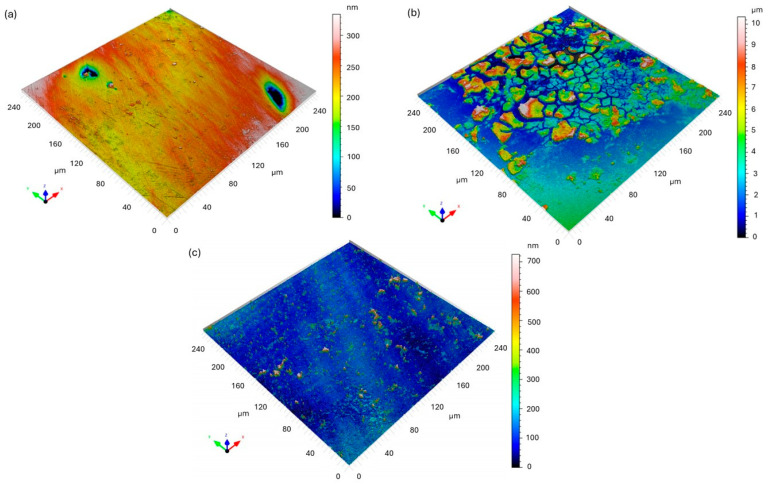
Three-dimensional confocal surface topography maps of Fe40Al5Cr0.2ZrB alloy: (**a**) before corrosion; (**b**) after corrosion—area 1 (intensive corrosion region); (**c**) after corrosion—area 2 (localized corrosion region).

Figure 5 presents the 3D surface topography of the Fe40Al5Cr0.2ZrB intermetallic alloy before and after exposure to a 5% NaCl solution, obtained using confocal microscopy and analyzed according to ISO 25178 parameters [24] are presented in Table 5 and Table 6. Post-corrosion observations were conducted at two locations on the sample.

Before corrosion (Figure 5a), the Fe40Al5Cr0.2ZrB surface exhibited a relatively smooth morphology characterized by a low arithmetic roughness (*S_a_* = 24.18 nm) and root mean square roughness (*S_q_* = 37.26 nm). The negative skewness (*S_sk_* = −1.618) and high kurtosis (*S_ku_* = 13.04) indicate that the surface was dominated by a few deep valleys, typical of mechanically polished metallic surfaces. No corrosion-related features were observed at this stage.

After immersion in the NaCl solution, significant changes in the surface morphology were detected. The first analyzed area (Figure 5b) showed a substantial increase in surface roughness (*S_a_* = 1.633 µm; *S_q_* = 2.082 µm) and total height (*S_z_* = 10.35 µm), evidencing intensive corrosion processes. The positive skewness (*S_sk_* = 1.005) and high surface development ratio (*S*_dr_ = 103.6%) indicate the formation of corrosion products and surface irregularities. This suggests the presence of localized corrosion phenomena leading to the accumulation of oxides and hydroxides on the Fe40Al5Cr0.2ZrB surface.

In contrast, the second analyzed region after corrosion (Figure 5c) demonstrated lower average roughness (*S_a_* = 41.28 nm; *S_q_* = 76.20 nm), but exceptionally high S_sk_ (4.544) and S_ku_ (32.19) values, indicating the presence of sharp, isolated peaks corresponding to localized pitting corrosion. This non-uniform topography confirms that the corrosion process on Fe40Al5Cr0.2ZrB is highly heterogeneous and may proceed through the formation of discrete anodic sites.

Overall, the confocal microscopy analysis revealed that corrosion in Fe40Al5Cr0.2ZrB alloy immersed in a 5% NaCl environment leads to both general surface degradation and local pitting. The drastic increase in *S_a_* and *S_z_* values, together with the change in *S_sk_* from negative to positive, confirms a transition from a smooth, valley-dominated surface to a rough morphology characterized by the accumulation of corrosion products and pit formation.

**Figure 6:**
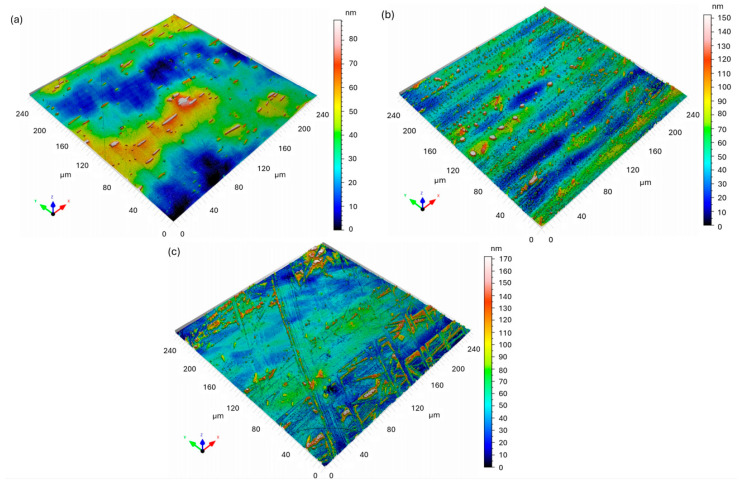
Three-dimensional confocal surface topography maps of X18CrN28 steel: (**a**) before corrosion; (**b**) after corrosion—area 1 (intensive corrosion region); (**c**) after corrosion—area 2.

Figure 6 shows the 3D confocal topography maps of X18CRN28 steel before and after exposure to a 5% NaCl solution. The surface parameters were determined in accordance with ISO 25178.

Before corrosion (Figure 6a), the X18CRN28 steel exhibited a smooth surface with low roughness values (S_a_ = 13.26 nm, S_q_ = 16.34 nm) and a moderate positive skewness (S_sk_ = 0.5556). The S_ku_ value of 3.244 indicates a nearly Gaussian height distribution with only minor surface asperities, which corresponds to a polished metallic surface without visible corrosion features.

After the corrosion test, the surface morphology changed only slightly compared to the Fe40Al5Cr0.2ZrB alloy. The first analyzed region (Figure 6b) showed a moderate increase in surface parameters (S_a_ = 16.30 nm, S_q_ = 22.57 nm, S_z_ = 152.4 nm). The positive S_sk_ (1.151) and relatively high S_ku_ (6.156) indicate the formation of isolated surface peaks, likely due to the initiation of local corrosion attack or the presence of small corrosion products.

The second region (Figure 6c) revealed slightly higher roughness (Sa = 18.89 nm, S_q_ = 26.67 nm, S_z_ = 171.9 nm) and S_sk_ = 1.373, confirming localized surface changes. The *S*_dr_ parameter (0.16%) remained very low, indicating that the surface did not undergo extensive degradation. Overall, the morphology of X18CRN28 remained relatively stable after immersion in a NaCl solution, exhibiting only minor roughening and isolated corrosion points.

Compared with the Fe40Al5Cr0.2ZrB alloy, the X18CrN28 steel exhibited superior corrosion resistance, as reflected by its much smaller increase in roughness and the absence of extensive corrosion-induced topographical changes. This finding is consistent with the higher passivity of Cr–Ni–containing steels and their ability to maintain a protective oxide layer in chloride-containing environments.

### 3.3. Atomic Force Microscopy (AFM)

Atomic force microscopy was applied to characterize the nanoscale surface morphology of the Fe40Al5Cr0.2ZrB alloy (sample 1) before and after corrosion testing in 5% NaCl solution. All measurements were conducted in intermittent contact mode on areas of 10 × 10 µm, followed by standard data-processing procedures to ensure accurate height and roughness analyses. Five independent regions per condition were examined to ensure statistical reliability.

**Figure 7:**
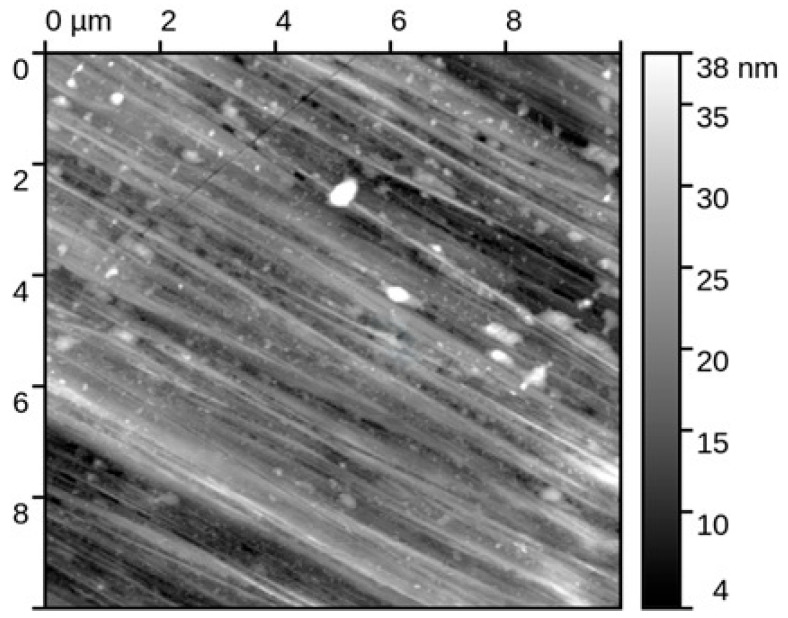
Topography of the original sample (Fe40Al5Cr0.2ZrB alloy) for areas of size 10 × 10 mm.

Surface condition before corrosion (Figure 7): The initial state of the Fe40Al5Cr0.2ZrB surface exhibited very low roughness values, with S_q_ = 1.65 ± 0.05 nm and S_a_ = 1.29 ± 0.17 nm, confirming a highly uniform, polished morphology typical for homogenized intermetallic materials. The observed nanoscale smoothness indicates the presence of a continuous surface oxide film formed during sample preparation, yet without corrosion-related features. This baseline surface condition provides a critical reference for evaluating corrosion-induced degradation.

**Figure 8:**
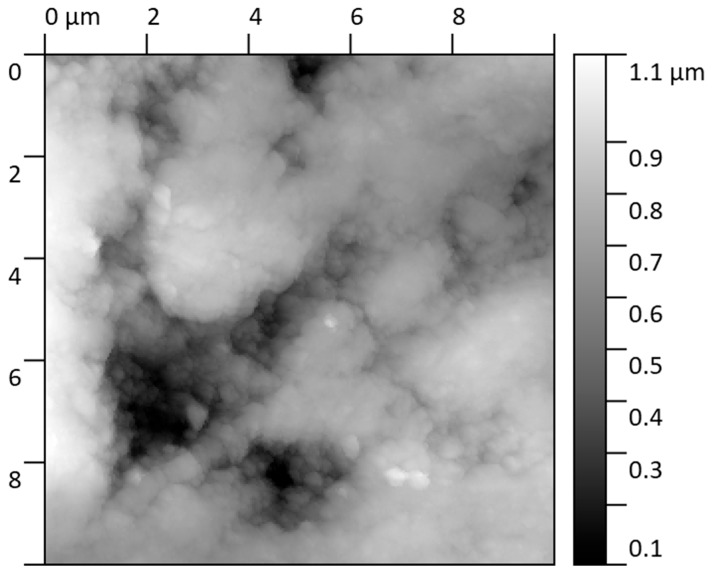
Topography of the sample (Fe40Al5Cr0.2ZrB alloy) in the corrosion zone covering an area of size 10 × 10.

The surface condition after corrosion (Figure 8) shows that exposure to a chloride solution induces a substantial modification of the nanoscale surface topography. Due to the strongly heterogeneous nature of the corrosion attack, roughness analysis could only be performed on the remaining unaltered regions of the specimen. In these selected locations, the S_q_ and S_a_ parameters slightly increased compared to the pristine state, reaching S_q_ = 1.89 ± 0.20 nm and S_a_ = 1.54 ± 0.11 nm, respectively. These values indicate that portions of the native surface remained passivated, maintaining nanoscale integrity.

However, the predominant surface transformation involved the formation of elevated morphologies in the corroded regions. These corrosion-generated structures can be described as plateau-like features with sharply defined boundaries, rising distinctly above neighboring unaffected areas. Quantitative profiling revealed a mean height of these “corrosion elevations” of 20.07 ± 2.56 nm, with individual profiles showing well-defined step geometries, indicating preferential local oxidation and accumulation of corrosion products. The AFM line profiles demonstrate abrupt transitions between intact and corroded domains, confirming susceptibility to localized attack mechanisms.

The interpretation of the corrosion mechanism and AFM observations support the electrochemical and SEM/EDS findings:

- Corrosion proceeds via localized breakdown of the passive Al-rich surface layer.
- Chloride ions promote selective formation of nanoscale corrosion product islands, consistent with early-stage pitting initiation.
- The coexistence of unaffected and severely altered nanoscale regions confirms competition between passivation and active dissolution pathways.

This behavior is characteristic of Fe40Al5Cr0.2ZrB intermetallics under chloride exposure, where mechanical or chemical defects in the oxide film act as nucleation points for aggressive attack, while adjacent locations remain temporarily passive.

**Figure 9:**
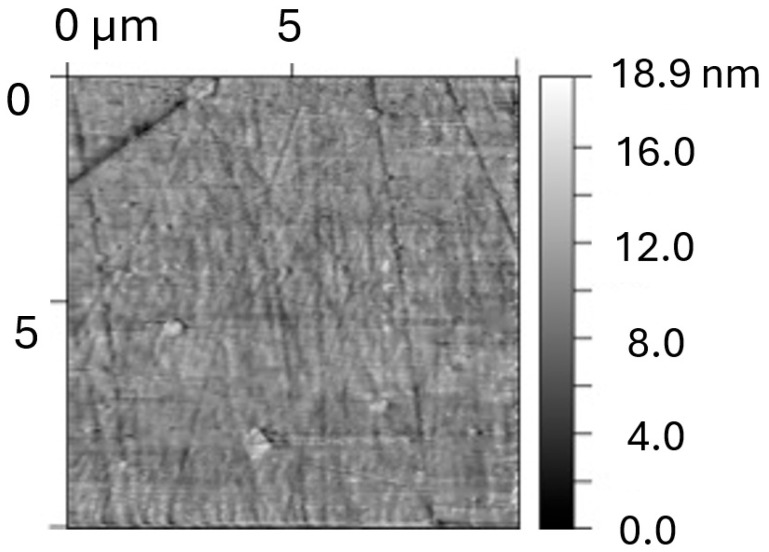
Topography of the original sample (X18CrN28 steel) (height) for areas of size 10 × 10 mm.

**Figure 10:**
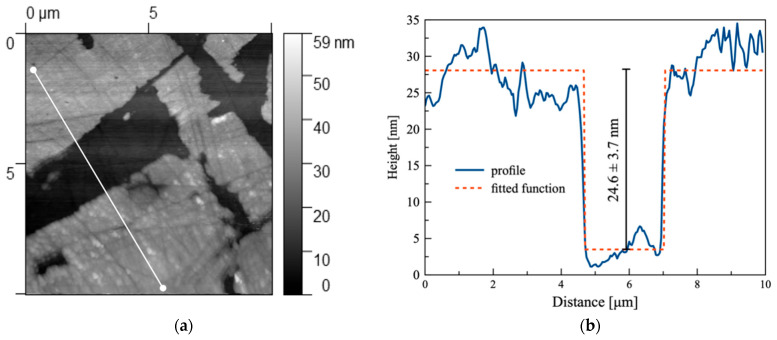
(**a**) Topography of the sample (X18CrN28 steel) in the corrosion zone covering an area of size 10 × 10. The white line indicates the position along which the height profile was extracted; (**b**) profile graph of the sample along the line marked in image (**a**); the dashed line represents the fitting function determined using the Gwyddion program (procedure: Fit critical dimension, function: Step height negative).

Unlike stainless steel X18CrN28 (Figure 9 and Figure 10), which was examined elsewhere in this study and retained a relatively smooth surface after corrosion exposure, Fe40Al5Cr0.2ZrB displayed a pronounced loss of morphological integrity already at the nanoscale level. The passive protection provided by alumina on FeAl alloys is insufficiently stable against chloride-driven breakdown, making these materials more vulnerable than high-Cr ferritic steels in saline aqueous environments.

### 3.4. Analysis of the Surface Condition After Corrosion Tests (SEM, EDS)

**Figure 11:**
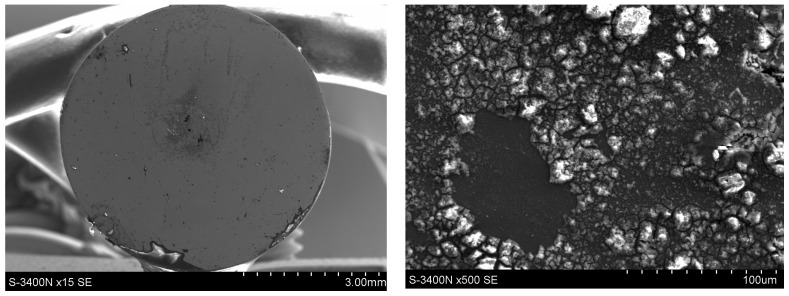
Surface condition of the Fe40Al5Cr0.2ZrB alloy after corrosion tests in 5% NaCl.

**Figure 12:**
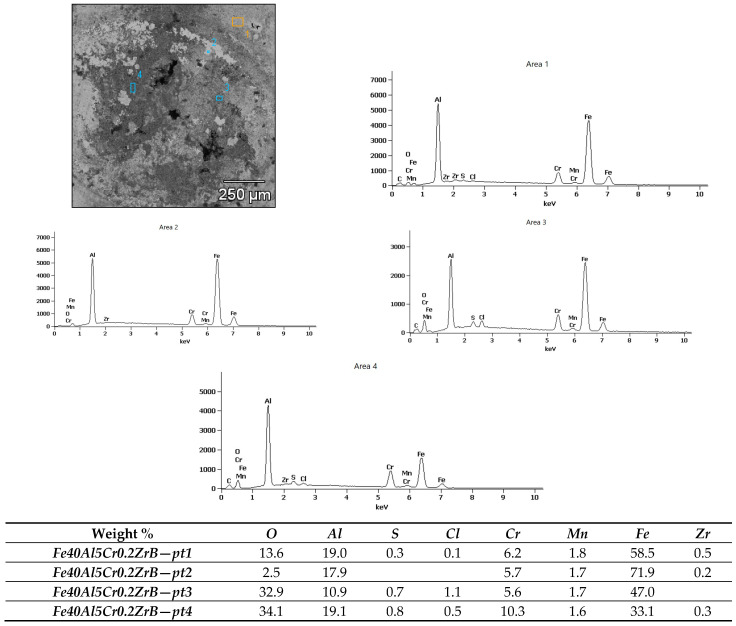
X-ray microanalysis of the chemical composition of corrosion products after testing in 5% NaCl of the Fe40Al5Cr0.2ZrB alloy.

Microscopic examinations (SEM) revealed pronounced differences in the surface condition of the two materials after exposure to a 5% NaCl solution. The surface of the Fe40Al5Cr0.2ZrB alloy (Figure 11) exhibited numerous corrosion products with heterogeneous morphology, with clearly visible regions of intense oxidation. The sharply defined depressions visible on the surface indicate the presence of pitting corrosion, which is consistent with the elevated levels of Cl and O detected in the EDS measurements (Figure 12). The most advanced forms of localized attack occur in regions that likely correspond to casting-related defects or microstructural discontinuities, which serve as preferential sites for pit initiation following the breakdown of the non-uniform Al_2_O_3_ layer. The observed morphology—comprising raised islands of corrosion products surrounded by deeper point-like cavities—indicates a strongly heterogeneous degradation process and local penetration of the oxide film.

**Figure 13:**
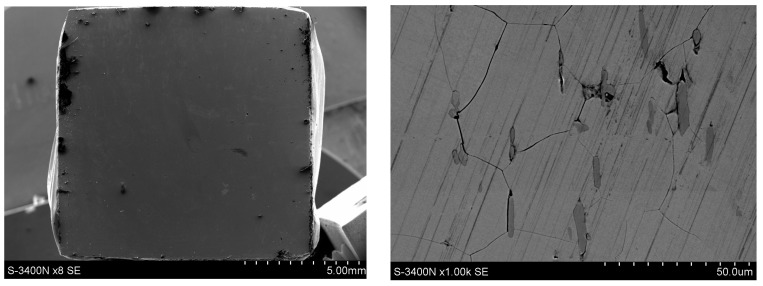
Surface condition of the X18CrN28 steel after corrosion tests in 5% NaCl.

**Figure 14:**
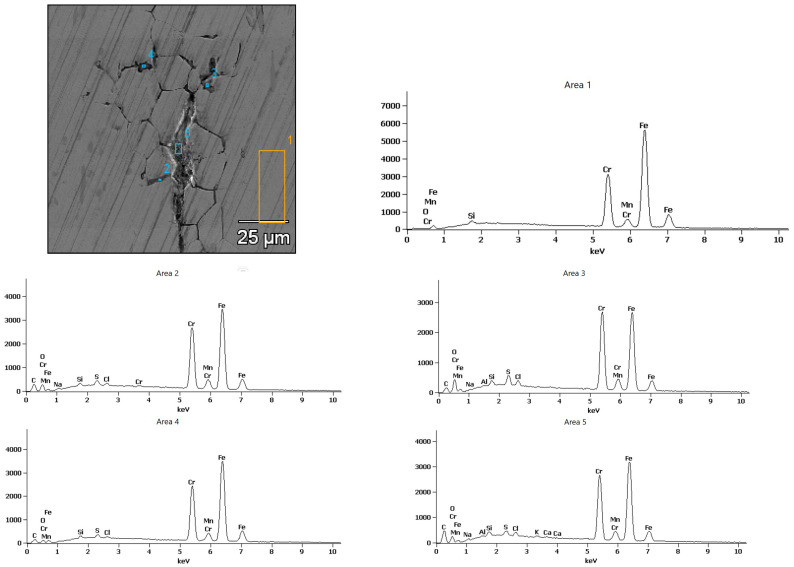
X-ray microanalysis of the chemical composition of corrosion products after testing in 5% NaCl of the X18CrN28 steel.

In contrast, the surface of the X18CrN28 steel (Figure 13) remained relatively smooth, with a uniformly distributed layer of protective oxides, indicating effective passivation in the chloride environment. However, locally found linear surface discontinuities are indicative of intergranular corrosion, which progresses along ferrite grain boundaries. This interpretation is supported by the EDS results shown in Figure 14, where the points corresponding to these linear features exhibit chromium enrichment, indicating the presence of chromium carbides of the Cr_23_C_6_ type. These carbides locally reduce the chromium content in the matrix and promote intergranular attack. It should be emphasized that such degradation features do not occur uniformly across the entire surface but appear only in selected, limited areas, consistent with the microscopic observations and indicating that the breakdown of the Cr_2_O_3_ passive film is partial rather than extensive.

Micro-area EDS analysis (Figure 12) confirmed substantial spatial variation in the chemical composition of the corrosion products on Fe40Al5Cr0.2ZrB. At the measurement points, oxygen ranged from 2.5 to 34.1%, aluminum 10.9–19.1%, iron 33.1–71.9%, and chromium 1.7–10.3%. The high oxygen contents at points 3 and 4 (over 30%) indicate an intensified oxidation process and the formation of an oxide layer, likely comprising a mixture of Al_2_O_3_, Fe_2_O_3_, and Cr_2_O_3_. The aluminum fraction within this layer points to the formation of protective Al_2_O_3_ oxides that partially limit further corrosion.

The detection of chlorine up to 5.7% (Fe40Al5Cr0.2ZrB-pt2) suggests localized penetration of the chloride solution and the initiation of pitting corrosion. The simultaneous presence of sulfur in small amounts (0.3–0.8%) may result from the adsorption of impurities or the formation of iron sulfides. Areas with higher iron and lower oxygen contents (e.g., Fe40Al5Cr0.2ZrB-pt2, 71.9% Fe) correspond to surface regions less covered by corrosion products.

For X18CrN28 steel, EDS analysis (Figure 14) indicates the dominance of alloying elements—iron, chromium, and nickel—with a markedly lower oxygen fraction compared to Fe40Al5Cr0.2ZrB. The corrosion products form a thin, continuous layer of Cr_2_O_3_ and FeCr_2_O_4_, confirming high passive-film stability. The absence of distinct chlorine peaks in the EDS spectra suggests that Cl^−^ ions do not significantly penetrate the passive layer.

Overall, X18CrN28 steel exhibits markedly superior corrosion resistance in the chloride environment compared to the Fe40Al5Cr0.2ZrB alloy. Although the high aluminum content in Fe40Al5Cr0.2ZrB favors the formation of Al_2_O_3_, the irregular structure and defects of this layer do not provide complete protection against Cl^−^ ingress.

## 4. Analysis of the Results

A comparative assessment of the corrosion resistance of a Fe40Al5Cr0.2ZrB intermetallic alloy and X18CrN28 ferritic stainless steel was carried out in 5% NaCl using EIS and potentiodynamic polarization, complemented by confocal/AFM imaging and SEM/EDS.

The corrosion resistance of potential replacement materials is typically evaluated using conventional electrochemical methods, such as potentiodynamic polarization curves and electrochemical impedance spectroscopy [25]. It was ascertained that the corrosion resistance of the X18CrN28 steel was significantly higher than that Fe40Al5Cr0.2ZrB alloy. Topographical and microanalytical observations indicated heterogeneous chloride attack in the Fe40Al5Cr0.2ZrB alloy (local pitting and surface roughening), while the steel showed only minor surface changes. The convergence of electrochemical and surface analyses enables an unambiguous ranking of the materials with respect to susceptibility to corrosion in NaCl solution. Consequently, X18CrN28 offers higher operational reliability in neutral chloride environments than the investigated Fe40Al5Cr0.2ZrB alloy. In the literature, it is well established that small additions of reactive elements, such as Zr and B, to Fe–Al intermetallic alloys primarily act as microstructural and scale-adhesion modifiers under high-temperature oxidizing conditions, rather than as effective inhibitors of aqueous corrosion. Studies on Fe–40Al alloys doped with Hf, Zr, and B have demonstrated that these elements promote the formation of a more adherent and continuous α-Al_2_O_3_ scale with parabolic growth kinetics and reduced spallation during thermal cycling [13,26]. Similarly, investigations of FeAl and FeCrAl alloys with Zr additions reveal that the presence of ZrO_2_ within the oxide scale enhances its adherence and mechanical stability, thereby improving oxidation resistance in the 900–1200 °C range [3]. Boron, on the other hand, exhibits a strong tendency to segregate to grain boundaries, increasing their cohesion and reducing intergranular brittleness in ordered B2 intermetallics such as FeAl [27,28]. In the context of the present work, Zr and B in the Fe40Al5Cr0.2ZrB alloy may therefore improve the stability and integrity of the alumina scale under high-temperature dry oxidation (as supported by previous studies on this alloy), but their influence in an aqueous chloride environment is limited. Neither Zr nor B prevents chloride adsorption onto the hydrated Al_2_O_3_ surface or chloride penetration through defects in the scale, which correlates with the observed pitting-type, heterogeneous corrosion morphology of Fe40Al5Cr0.2ZrB in 5% NaCl. Unlike the high chromium content in X18CrN28 steel, which supports the formation of a stable, self-healing Cr_2_O_3_-based passive film resistant to chloride attack, the role of Zr and B in the Fe–Al alloy is mainly that of a “high-temperature” scale stabilizer rather than an enhancer of electrochemical corrosion resistance in saline aqueous media.

Despite the significant differences in the initial microstructure—namely the single-phase, ordered B2 structure of the Fe40Al5Cr0.2ZrB alloy and the ferritic microstructure of the X18CrN28 steel with a homogeneous distribution of chromium—the decisive factor governing their corrosion behavior in 5% NaCl is not the microstructure itself but the fundamentally different physicochemical mechanisms responsible for passive film formation and stability. In the Fe–Al alloy, the passive film formed under the tested conditions consists of a thin Al_2_O_3_ layer exhibiting numerous surface defects and a columnar oxide structure, which facilitates local electrolyte penetration [29]. Defects and grain boundaries within the Al_2_O_3_ film act as preferential sites for chloride adsorption; atomistic studies have demonstrated that Cl^−^ ions can substitute surface −OH groups on hydroxylated alumina and initiate localized breakdown by forming unstable Al–O–Cl surface complexes [15,30,31]. This leads to pointwise depassivation and pit initiation. The slow repassivation kinetics and the limited ability to restore a compact Al_2_O_3_ layer promote the development of heterogeneous, isolated active sites, as confirmed by SEM/EDS observations (local enrichment in Cl and O), as well as 3D topography and AFM analyses, which show raised corrosion-product regions surrounding active dissolution zones.

In contrast, the X18CrN28 steel forms a stable, compact, and low-defect Cr_2_O_3_-based passive film due to its high chromium content. Recent studies show that increasing Cr content in ferritic steels enhances the formation of passive films with lower ionic conductivity and fewer structural defects, thereby improving resistance to chloride penetration and facilitating rapid repassivation [32,33]. The Cr_2_O_3_ film additionally exhibits high thermodynamic stability in chloride-containing environments and does not readily form volatile or unstable Cr–O–Cl compounds, limiting the initiation of surface defects [34]. As a result, corrosion proceeds in a mild and dispersed manner, and the surface topography remains almost unchanged—consistent with the minimal increases in S_a_ and S_z_ parameters and the absence of pronounced pitting in SEM images.

Therefore, despite the microstructural differences between the two materials, the key factor determining their corrosion performance in NaCl solution is the chemical nature and stability of their passive films—a defect-prone and chloride-susceptible Al_2_O_3_ film in Fe40Al5Cr0.2ZrB versus a stable, repassivable Cr_2_O_3_-based film in X18CrN28 steel. These physicochemical differences, rather than microstructural ones, underpin the markedly different degradation mechanisms observed in both materials.

## 5. Conclusions

Based on the conducted tests and analysis of their results, the following conclusions were formulated:

1. X18CrN28 steel is characterized by more favorable corrosion parameters than the Fe40Al5Cr0.2ZrB alloy.
2. Confocal/AFM and SEM/EDS confirm heterogeneous degradation of the Fe40Al5Cr0.2ZrB alloy and the preservation of a thin, continuous passive film on the steel.
3. The material ranking derived from electrochemical methods is consistent with topographical and corrosion-product analyses.
4. For chloride-exposed applications, X18CrN28 steel is recommended; for Fe40Al5Cr0.2ZrB alloys, barrier coatings or surface modifications should be considered.

## Figures and Tables

**Table 1:** Chemical composition of Fe40Al5Cr0.2ZrB alloy [6].

Compound	Fe	Al	Cr	Zr	B
% at.	54.80	40.10	4.86	0.18	0.06

**Table 2:** Chemical composition of X18CrN28 steel.

Compound	C	Mn	Si	Cr	Ni	N	Fe
% mas.	0.15	0.72	0.36	23.70	0.25	0.15	rest

**Table 3:** EIS parameters determined for the tested materials.

Type of Materials	*R*_s_ [Ω·cm^2^]	*T*	*ϕ*	*R*_p_ [Ω·cm^2^]
Fe40Al5Cr0.2ZrB alloy	1.89	0.00001799	0.89	12,330
X18CrN28 steel	1.48	0.00001013	0.77	49,230

**Table 4:** Corrosion parameters: *E*_corr_, *j*_corr_ and *R*_p_ of the tested materials.

Type of Materials	*E*_corr_ [V]	*j*_corr_ [μA·cm^−2^]	*R*_p_ [Ω·cm^2^]
Fe40Al5Cr0.2ZrB alloy	−0.502	3.69	12,790
X18CrN28 steel	−0.223	0.02	49,670

**Table 5:** Parameters describing the surface topography of the Fe40Al5Cr0.2ZrB alloy.

Sample/Parameter	S_a_	S_q_	S_sk_	S_ku_	S_z_
**Sample before corrosion test**	24.18 nm	37.26 nm	−1.618	13.04	334.8 nm
**Sample after corrosion test, area 1**	1.633 µm	2.082 µm	1.005	3.809	10.35 µm
**Sample after corrosion test, area 2**	41.28 nm	76.20 nm	4.544	32.19	725.3 nm

**Table 6:** Parameters describing the surface topography of the X18CrN28 steel.

Sample/Parameter	S_a_	S_q_	S_sk_	S_ku_	S_z_
**Sample before corrosion test**	13.26 nm	16.34 nm	0.5556	3.244	88.46 nm
**Sample after corrosion test, area 1**	16.30 nm	22.57 nm	1.151	6.156	152.4 nm
**Sample after corrosion test, area 2**	18.89 nm	26.67 nm	1.373	5.966	171.9 nm

## Data Availability

The original contributions presented in this study are included in the article. Further inquiries can be directed to the corresponding authors.
